# Editorial: Social determinants of women’s health in low and middle income countries

**DOI:** 10.3389/fgwh.2024.1482047

**Published:** 2024-10-28

**Authors:** Rubeena Zakar, Sarosh Iqbal

**Affiliations:** ^1^ Department of Public Health, University of the Punjab, Lahore, Pakistan; ^2^ Department of Sociology, School of Social Sciences & Humanities, University of Management & Technology, Lahore, Pakistan

**Keywords:** social determinant, women’s health, LMIC (low- and middle-income countries), peripartum, maternal health

**Editorial on the Research Topic**
Social determinants of women's health in low and middle income countries

The recognition of women's improved health and addressing health inequities is critical to achieving the Sustainable Development Goals. The Social Determinants of Health (SDOH) provide a vital framework to comprehend and interpret health disparities, particularly among women in low- and middle-income countries (LMICs). The social determinants of women's health are multidimensional, encompassing political, socio-cultural, economic, and environmental factors that extend beyond bio-medical causes (1, 2).

A holistic approach to SDOH emphasizes the influence of political ecology (3) and various social structures impact women's health in LMICs. It calls for an integrated analysis of the social and environmental systems and policies, which restrict women from opting for healthier opportunities leading to increased maternal mortality and morbidity (3). Socio-economic inequities—such as poverty, limited resources, and lack of education disproportionately affect maternal and children's health outcomes (4, 5). Deeply entrenched patriarchal structures, cultural practices, gender norms further exacerbate these disparities by restricting women's decision-making power (3, 6, 7). The intersection of gender with race, ethnicity, and socioeconomic status further widens these gaps leading to poorer health indicators of marginalized women (4). Further, systemic biases, unfamiliarity with health care systems, and social injustices also contribute to poor maternal health outcomes (8).

The SDOH framework highlights the need for an equity-focused approach, shifting beyond a biomedical model of healthcare to address women's health disparities and create an enabling environment for their well-being (9). This collection of 13 original research articles from LMICs highlights how various social determinants influence maternal health. These comprised of eight manuscripts from Ethiopia, such as two secondary analyses of the Demographic and Health Survey (DHS) (Belay et al., Mebratie), three cross- sectional research studies (Deresa Dinagde et al., Tolesa et al., Desta et al.), two community-based case-control studies (Teressa et al., Aleye et al.), and mixed- method research (Dibaba Degefa et al.). Further, there is a qualitative research from India (Ahuja et al.), an empirical analysis of Papua New Guinea (Shen et al.), a prospective study from Ghana (Tettegah et al.), and a cohort study from Tanzania (Mbwele et al.). Furthermore, there is a historical cohort study from 6 countries, i.e., Democratic Republic of Congo, Guatemala, India, Kenya, Pakistan, and Zambia (Nyongesa et al.). In sum, these thirteen original research articles shed light on three major intersecting themes shaping the social determinants of women's health in LMICs: socio-economic dynamics, socio-cultural norms, and structural and environmental conditions.

## Socio-economic dynamics

Predominately, this collection indicated the crucial role of socio-economic dynamics influencing women’ health during the prepartum, intrapartum, and postpartum periods. These factors included lower literacy rates, geographies and residential disparities (rural vs. urban), poor economic conditions, occupational status, and lack of decision-making autonomy. For instance, the collection included two secondary analyses of the 2016 Ethiopian DHS (Belay et al., Mebratie), underscored socio-economic disparities. In a study by Mebratie on antenatal care (ANC) and its associated factors, it was found that mothers from rural areas, with lower educational levels, and belonging to the lowest wealth quintile were significantly more marginalized in receiving essential ANC compared to their counterparts in the highest wealth quintiles in Ethiopia. Similarly, Belay et al. found that women's education, geographical location, wealth index, mass media exposure, unintended pregnancy, and multi-parity were strong predictors of the first ANC visit in Ethiopia.

Women from disadvantaged socio-economic backgrounds are more vulnerable to health risks. A prospective study by Tettegah et al. in Ghana demonstrated that exposure to risk factors, including lower education and occupational status of pregnant women, increased their susceptibility to anemia, even when they attended ANC.

Socioeconomic contexts generally shape women's social status in a society's hierarchy. Recognizing the link between women's social status, empowerment, and maternal and child health services, Shen et al. evaluated the causal relationship between these factors and skilled birth attendance using Papua New Guinea DHS (2016–18). This study revealed that women's education substantially improved their decision-making autonomy, positively contributing to maternal healthcare utilization in low-resource settings.

These studies underscore the importance of educating and empowering women by improving their socioeconomic status and addressing disparities to enhance maternal healthcare utilization in LMICs.

## Socio-cultural norms

Prevailing socio-cultural norms surrounding pregnancy and childbirth outline maternal health behaviors and healthcare-seeking practices in LMICs, including maternal age and autonomy to start a family, and women's choices regarding nutrition and childbirth practices. Aleye et al. conducted a community-based case-control study in Eastern Ethiopia and found that maternal age influenced peripartum care utilization, leading to shorter birth intervals. Similiarly, Nyongesa et al., using a historical cohort (2010–2020), analyzed maternal and perinatal outcomes based on maternal age across six low-resource settings, including the Democratic Republic of Congo, Guatemala, India, Kenya, Pakistan, and Zambia. The increased prevalence of adolescent pregnancies in these settings reflected early marriages and pregnancies in, consistent with local cultural practices. These early pregnancies often occur in environments where girls face cultural restrictions, limited opportunities, gender inequality, and poverty, which not only heighten the risk of adverse perinatal outcomes but also perpetuate cycle of disadvantage.

Conversely, while advanced maternal age pregnancies were less common, they were associated with a higher risk of maternal and perinatal adverse outcomes. This finding suggests that women in deprived circumstances may delay childbirth due to economic instability and a lack of resources for child-rearing.

Additionally, Tettegah et al. assessed the hemoglobin levels of pregnant women in Ghana and identified associated risk factors. Despite greater awareness of anemia, cultural norms limited access to nutritious diets and medical care during pregnancy. These findings suggest that a lack of women's health education about nutritious food during pregnancy, coupled with cultural customs, increases the risk of anemia. Similarly, Ahuja et al. observed dietary restrictions in India due to strong cultural beliefs and practices during pregnancy and the postnatal period. Their study highlighted how these practices negatively impacted maternal and infant health, leading to postpartum depression and isolation, which requires urgent attention.

Gender roles, societal expectations and cultural norms generally affect women’ health education, their informed decision making capacity and adoption of healthier practices in LMICs. This is evident from a community-based unmatched case-control study by Teressa et al. in Southern Ethiopia. The results highlighted that inadequate health education and poor maternal healthcare utilization often lead to home deliveries, increasing the risk of maternal mortality or morbidity, especially for marginalized women. Health education can be a catalyst, empowering women during the peripartum period to recognize the benefits of institution-based deliveries for the well-being of both mothers and newborns.

Socio-cultural beliefs arising from SDOH highly influence factors affecting women by exacerbating pre-existing conditions, with serious implications on maternal health outcomes. The vulnerability of socio-economic status coupled with traditional practices often result in poor nutritional intake and unhealthy dietary practices, increasing the risk for anemia and other maternal complications. Any deficiency or health risk during pregnancy or childbirth can have detrimental effects on both fetus and the mother.

## Structural and environmental conditions

Structural and environmental inequities often lead to adverse maternal outcomes when disparities in access, resource distribution and power dynamics are reinforced through structural mechanisms (10). Marginalized women in LMICs experience multiple layers of inequalities in healthcare system, influencing both their access to and utilization of quality maternal healthcare services. Beyond the health system, other structural factors, such as governance, social and public policies, and social values (11), contribute to variations in social determinants of health (SDOH) and shape women's healthcare opportunities. This collection primarily focused on maternal healthcare access and utilization during the perinatal period, particularly ANC and PNC services, highlighting barriers and inadequacies in healthcare infrastructure. However, it lacked an appraisal of the enforcement of existing policies and programs designed to enhance these services.

Deresa Dinagde et al. conducted an institution-based cross-sectional study that assessed optimal ANC utilization among pregnant women. It revealed that only less than half of the pregnant women achieved optimal ANC in Southern Ethiopia. Similarly, Mebratie found lower levels of awareness and utilization of core ANC components, particularly among women from rural areas. Subsequently, a mixed-method research by Dibaba Degefa et al. estimated more than 55% PNC utilization among women in Southern Ethiopia. Overall, a significant proportion of women in LMICs demonstrated a relatively unsatisfactory maternal healthcare utilization, with obstacle such as distant health facilities, long travel times, and extended waiting hours. There is a need to address these barriers related to access to maternal health service and promote health education on importance of regular check-ups for ANC and PNC service utilization, and awareness of pregnancy danger signs to prevent adverse maternal outcomes.

Maternal satisfaction with labor and delivery is an essential indicator of healthcare access and quality of care. Tolesa et al. assessed maternal satisfaction with intrapartum care in public hospitals in Ethiopia, finding that more than 50% of mothers were satisfied with the care they received, indicating healthcare access and quality. Another community-based cross-sectional survey by Desta et al. assessed maternal healthcare utilization was associated with women's perception of respectful treatment by service providers, with satisfaction towards health facility infrastructure being a key contributors to healthcare utilization. Additionally, Aleye et al. found that peripartum care utilization affected short birth intervals, suggesting that improving maternal healthcare utilization is indispensable to reducing the burden of short birth intervals.

Institutional deliveries reduce the likelihood of adverse maternal outcomes. Teressa et al. reported that inadequate health education, poor maternal healthcare utilization, and resistance to institutional deliveries result in home births, thus may increase the risk of maternal complications, maternal mortality and morbidity.

Lastly, Mbwele et al. highlighted the role of healthcare providers and measured the impact of blended training on obstetric hemorrhage management in Tanzania. The training was found to significantly improve healthcare providers’ knowledge and skills, emphasizing the potential of blended training in reducing maternal deaths.

This research agenda on social determinants of women's health largely contributes to the existing literature, providing valuable insights for academics, researchers, program managers/implementers, and policymakers. It encapsulates a broader spectrum of SDOH influencing maternal and children's health and addressing health disparities in systemic and meaningful ways to promote a more equitable society.

Key lessons include the need for addressing systemic barriers such as inadequate healthcare infrastructure, unequal resource distribution, and restrictive cultural practices that limit women's access to and utilization of maternal healthcare services. Health education, particularly regarding antenatal and postnatal care, can empower women and improve health-seeking behaviors, while enhancing governance and enforcing policies are critical to improving healthcare access. Future research should focus on the intersectionality of social determinants, specifically how socio- economic status, cultural practices, and gender inequalities impact maternal health outcomes. Additionally, there is a need to evaluate and implement targeted interventions, such as blended training for healthcare providers and community-based health education programs, to mitigate these challenges and ensure equitable healthcare access for women in LMICs. By focusing on these areas, research can contribute to developing sustainable strategies that promote women's empowerment and improve maternal and child health in resource-constrained settings.
